# Efficacy of Bone Marrow-Derived Stem Cells on Non-Ischemic Cardiomyopathy: A Systematic Review and Meta-Analysis of Randomized Controlled Trials

**DOI:** 10.3390/jcm14217610

**Published:** 2025-10-27

**Authors:** Tri Wisesa Soetisna, Fegita Beatrix Pajala, Harry Raihan Alzikri, Maasa Sunreza Millenia, Anwar Santoso, Erlin Listiyaningsih

**Affiliations:** 1 Adult Cardiac Surgery Division, Thoracic and Cardiovascular Surgery Department, National Cardiovascular Center Harapan Kita, Jakarta 11420, Indonesia; fegita_pajala@yahoo.com (F.B.P.); eanhary.md@gmail.com (H.R.A.); msunreza@gmail.com (M.S.M.); 2 Cardiology and Vascular Medicine Department, National Cardiovascular Center Harapan Kita, Jakarta 11420, Indonesia; awscip@gmail.com; 3 Faculty of Medicine, Prof. Dr. Hamka Muhammadiyah University, Jakarta 12130, Indonesia; erlin.soedarmo@uhamka.ac.id

**Keywords:** non-ischemic cardiomyopathy, stem cell therapy, bone marrow-derived stem cell therapy

## Abstract

**Background/Objectives**: Non-ischemic cardiomyopathy (NICM) refers to myocardial disease characterized by structural and functional impairment without coronary artery disease. Stem cell therapy has emerged as a potential treatment to restore heart function in NICM, but clinical results have been inconsistent. **Methods**: This meta-analysis comprises five randomized controlled trials with a total of 302 patients, retrieved from PubMed, ScienceDirect, the Cochrane Library, and SAGE Journals. **Results**: Compared with the control group, stem cell therapy group showed significant improvements in the left ventricular ejection fraction (LVEF) at the 3-month follow-up (MD = 4.55, 95% CI 2.12–6.98, *p* = 0.0002), a reduction in the left ventricular end-diastolic diameter (LVEDD) at the 3-month follow-up (MD = −3.83, 95% CI −7.27 to −0.39, *p* = 0.03) and an improvement in the New York Heart Association (NYHA) functional class both at 3 months (MD = −0.58 95% CI −0.97 to −0.19, *p* = 0.004) and 12 months (MD = −0.49 95% CI −0.91 to −0.07, *p* = 0.02). Additionally, there was a significant decrease in the Minnesota Living with Heart Failure Questionnaire (MLHFQ) score at the 6-month follow-up (MD = −14.05, 95% CI −25.97 to −2.13, *p* = 0.021). However, no significant differences were observed in the left ventricular end-diastolic volume (LVEDV), left ventricular end-systolic volume (LVESV), 6-min walk test (6-MWT), or major adverse cardiovascular events (MACEs) between the two groups. **Conclusions**: Bone marrow-derived stem cell therapy could be a promising and safe method to improve cardiac function and quality of life in patients with NICM. Further large-scale randomized controlled trials are needed to validate these findings.

## 1. Introduction

Non-ischemic cardiomyopathy (NICM) is a myocardial disease not associated with ischemic injury. It is classified into several subtypes based on ventricular morphology and functional characteristics assessed by cardiac imaging (echocardiography, CT-scan, or cardiac MRI). These subtypes included dilated cardiomyopathy (DCM), hypertrophic cardiomyopathy (HCM), restrictive cardiomyopathy (RCM), non-dilated left ventricular cardiomyopathy (NDLVC), and arrhythmogenic right ventricular cardiomyopathy (ARVC) [1]. The most frequent type of NICM, with a prevalence of 1 in 2500 individuals worldwide, is DCM. NICM causes 5.72 million disability-adjusted life years (DALYs) and around 240,000 deaths worldwide, according to data from the Global Burden of Disease (GBD) 2021. NICM profoundly affects quality of life and contributes substantially to long-term morbidity. Its burden differs across geographic regions. In moderate- to high-income countries, ischemic heart disease remains a major contributor to cardiovascular mortality. In contrast, Southern Sub-Saharan Africa has the highest NICM-related mortality rates, at 11.3 per 100,000 population [2,3,4].

Non-ischemic cardiomyopathy can be genetic or acquired. The pathophysiology of NICM involves a complex interaction of genetic, structural, inflammatory, and metabolic factors that lead to myocardial dysfunction without coronary artery disease. Genetic mutations are a major contributor, particularly in DCM, ARVC, and HCM, where sarcomeric defects, cytoskeletal, or desmosomal proteins disrupt normal myocardial architecture and function [2]. The MOGE(S) classification characterizes cardiomyopathies using five elements: M (morpho-functional phenotype), O (organ involvement), G (genetic or familial inheritance), E (etiological factors), and S (functional status). This system was introduced by the World Heart Federation (WHF) to provide a detailed and comprehensive framework for describing cardiomyopathies, integrating the strengths from both AHA and ESC classifications [5].

The aims of NICM treatment are to enhance cardiac function, relieve symptoms, and reduce complications such as heart failure or sudden cardiac death. Standard treatment consists of pharmacological and device-based therapies, which help the heart work more efficiently and minimize the risk of hospitalization or mortality [6,7]. Despite the advancement in medical and device therapy, a subset of patients with NICM fail to achieve significant benefits from guideline-directed medical therapy, with disease progression characterized by fibrosis and a five-year mortality rate of approximately 30% [8,9,10,11].

Stem cell therapy study in NICM is less common compared to ICM because the myocardial regeneration concept is more easily applied, as it usually presents with a well-defined area of myocardial loss and scar tissue, in contrast with NICM, which often involves inflammation that creates diffuse myocardial fibrosis [12]. However, evidence from head-to-head comparisons suggests that the response to stem cell therapy differs between NICM and ICM, but this is still poorly understood [13]. Although current medical and device-based treatments for NICM can reduce disease progression and improve quality of life, they do not directly treat the underlying myocardial damage and do not have the ability to regenerate heart tissue. Due to this limitation, there has been increasing interest in stem cell treatment as a possible means of replacing or repairing damaged cardiac tissue [14,15,16,17]. Our study investigates whether stem cells can promote myocardial regeneration, reduce fibrosis, and enhance cardiac function in patients with NICM.

## 2. Materials and Methods

The PROSPERO database prospectively registered this review protocol under the ID CRD420251128422.

### 2.1. Study Design

The Preferred Reporting Items for Systematic Reviews and Meta-Analyses (PRISMA) criteria were followed in the conduct of this meta-analysis [18]. Using randomized controlled trials (RCTs), the goal was to methodically assess the safety and effectiveness of stem cell therapy in individuals with non-ischemic cardiomyopathy (NICM).

### 2.2. Eligibility Criteria

Initial eligibility criteria were used to determine which studies were included in this evaluation. Enrolled eligible studies included the following: (1) adult patients (≥18 years) diagnosed with non-ischemic cardiomyopathy (NICM); (2) any form of stem cell therapy (e.g., bone marrow-derived, mesenchymal, or cardiac progenitor cells delivered via intracoronary, intramyocardial, or transendocardial routes; (3) comparator groups that received either placebo or standard medical therapy without stem cell intervention; (4) studies that reported at least one of the following outcomes: changes in the left ventricular ejection fraction (LVEF), left ventricular end-diastolic volume (LVEDV), left ventricular end-systolic volume (LVESV), New York Heart Association (NYHA) functional class, 6-min walk distance (6MWD), or adverse events; (5) only randomized controlled trials (RCTs); and (6) English-language studies. Research was not included if it (1) focused on ischemic cardiomyopathy or included mixed populations without separate NICM data, (2) was a case report, review, editorial, or conference abstract, or (3) had a follow-up period of less than three months or lacked quantitative outcome data.

### 2.3. Search Strategy

From its launch until August 2025, an in-depth literature review was carried out across five large databases: PubMed, ProQuest, Science Direct, Cochrane Library, and SAGE Journal. The appropriate search terms and MeSH phrases that were used in the search strategy were “Non-ischemic cardiomyopathy” OR “dilated cardiomyopathy” AND “stem cell therapy” OR “cell-based therapy” OR “regenerative therapy” AND “randomized controlled trial” OR “clinical trial”. To discover additional relevant studies that might have passed through the first search, a manual review of reference lists from all included studies and pertinent review articles may follow, in addition to the computerized database search.

### 2.4. Study Selection

The titles and abstracts of the studies that were retrieved were independently checked by two reviewers [FBP, HRA]. For studies that fulfilled the inclusion criteria or whose abstracts lacked adequate material, full-text papers were acquired. Discussions or contact with a third reviewer [AS] were used to settle disagreements over the selection of studies.

### 2.5. Data Extractions, Quality Assessment, and Variable Definition

Using standardized forms, two reviewers [MSM, EL] separately gathered data. The following data was gathered: publication details (corresponding author, year, country, and journal), study populations (age, sex, and eligibility criteria), intervention details (diagnosis, cell type, total number of cells injected (when available), route of administration, and number of cells administered), study design (sample size, methodology, and duration of follow-up), and clinical outcomes for efficacy and safety (e.g., major adverse cardiac events [MACEs]).

We adopted the Cochrane Risk of Bias-2 (RoB-2) tool to evaluate the risk of bias in the included randomized controlled trials (RCTs) [19]. Key methodologies such as random sequence generation, allocation concealment, participant and staff blinding, outcome assessment blinding, completeness of outcome data, and selective outcome reporting were all included in the evaluation criteria.

The main outcomes evaluated in this meta-analysis included improvements in New York Heart Association (NYHA) functional class, performance on the 6-min walk test (6-MWT), and changes in the left ventricular ejection fraction (LVEF), left ventricular end-diastolic diameter (LVEDD), left ventricular end-diastolic volume (LVEDV), and left ventricular end-systolic volume (LVESV), which are important measures of cardiac function and physical performance. These outcomes were chosen in order to assess gains in heart performance after intervention, both structurally and functionally. Secondary outcomes were quality of life, assessed by the Minnesota Living with Heart Failure Questionnaire (MLHFQ), and the number of MACEs, which served as a marker of overall safety and long-term clinical impact.

### 2.6. Statistical Analysis

Review Manager (RevMan) version 5.4 (The Cochrane Collaboration, Copenhagen) was utilized for pooled analysis. Weighted mean differences (WMDs) or standardized mean differences (SMDs) with 95% confidence intervals (CIs) were calculated for continuous variables (including LVEF, LEDV, LVESV, and LVEDD). The odds ratio (OR) was used for outcomes that were dichotomous (e.g., MACE). The I2 statistic was used to evaluate heterogeneity; I2 > 50% implies significant heterogeneity. When heterogeneity was significant, a random-effects model was employed; otherwise, a fixed-effects model was utilized. Using Egger’s test and funnel plots, publication bias was evaluated.

## 3. Results

**Figure 1:**
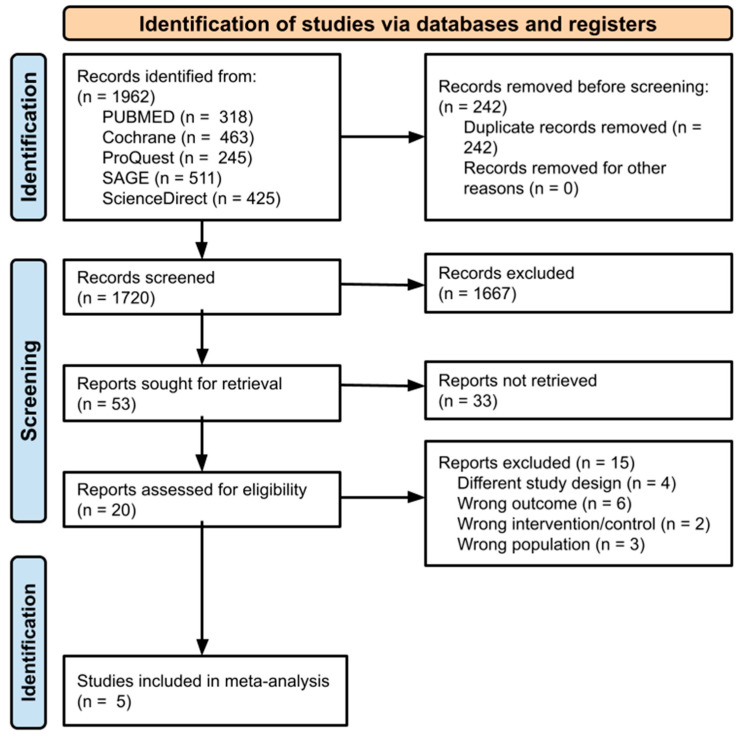
PRISMA 2020 flowchart diagram.

The process of study selection and the outcomes were illustrated in a PRISMA flow diagram as displayed in Figure 1. The initial search strategy resulted in 1962 potentially relevant studies. Following the elimination of duplicates, 1720 studies were left for screening of titles and abstracts. According to the inclusion criteria, 20 studies were selected for full-text review. Of these, four had different study designs, three featured the wrong outcome, two had the wrong intervention/control, and three involved the wrong population. Finally, five studies satisfied the requirements for data extraction and were incorporated into the meta-analysis.

### 3.1. Quality Assessment

**Figure 2:**
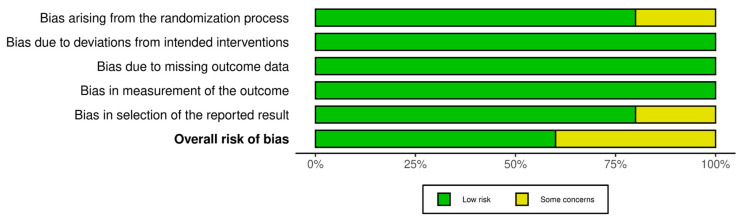
Summary plot of the quality assessment of the included RCTs.

**Figure 3:**
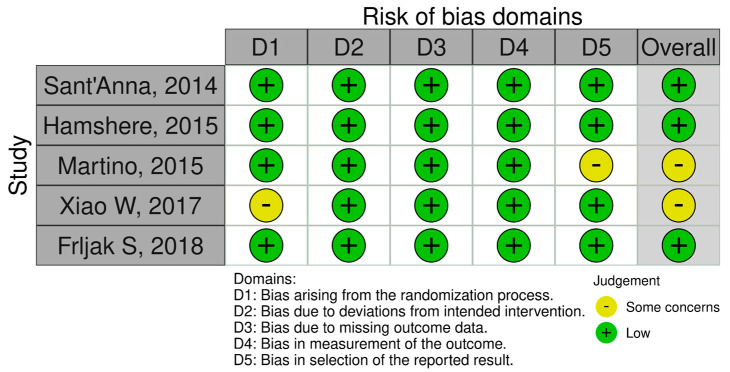
Traffic light plot of the quality assessment of the included RCTs.

The quality evaluation of the included studies was conducted using the Cochrane Risk of Bias Tool 2.0 (RoB 2) specifically for RCTs. Out of the five studies, three were categorized as having a low risk of bias, whereas two studies were classified as having a moderate risk of bias. The quality assessment results for all included studies are shown in Figure 2 and Figure 3.

### 3.2. Study Characteristics

Five studies involving a total of 302 participants were analyzed, including 143 patients in the intervention groups and 159 patients in the control groups. Out of five trials, two were conducted in Brazil, one in the UK, one in China, and one in the United States. The majority of participants were male (69.5%). Three studies used BMMC, one study used BMSC, and one study used CD34^+^ as the treatment group. Intracoronary stem cell injection was the most common route of stem cell administration across studies. The follow-up period ranged from 3 months to 12 months. The characteristics of the included studies are displayed in Table 1.

### 3.3. Meta-Analysis Results

#### 3.3.1. Left Ventricular Ejection Fraction

All studies reported the LVEF outcome with different follow-up periods. At the 3-month follow-up, the stem cell group exhibited a notable improvement in LVEF compared to the control group (MD = 4.55, 95% CI 2.12 to 6.98, *p* = 0.0002). The heterogeneity among studies was low (I^2^ = 0%, *p* = 0.56), indicating consistent results.

**Figure 4:**
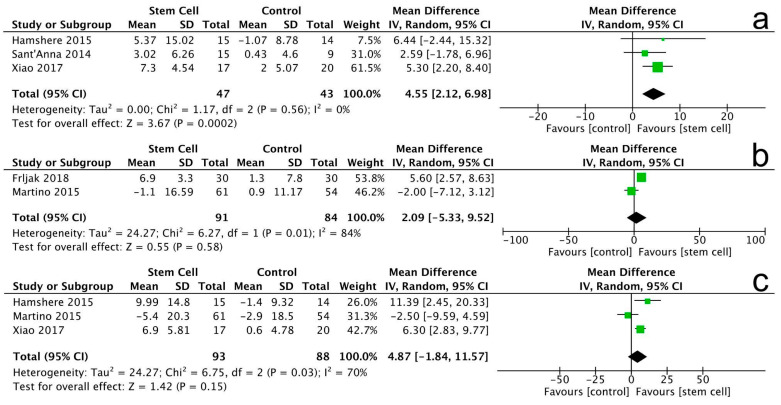
Forest plot for LVEF changes at 3 months (**a**), 6 months (**b**), and 12 months (**c**).

However, after the follow-up periods of 6 and 12 months, there were no significant differences between the stem cell group and the control group (MD = 2.09, 95% CI −5.33 to 9.52, *p* = 0.58) and (MD = 4.87, 95% CI −1.84 to 11.57, *p* = 0.15), respectively (Figure 4).

#### 3.3.2. Left Ventricular End-Diastolic Volume

**Figure 5:**
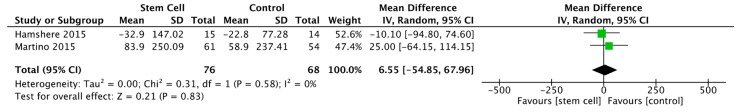
Forest plot for LVEDV changes at 12 months.

A total of 2 trials with 144 patients reported the association between the stem cell therapy group and the control group. At the 12-month follow-up, the stem cell group demonstrated a non-significant LVEDV improvement between the two groups (MD = 6.55, 95% CI −54.85 to 67.96, *p* = 0.83). The heterogeneity among the studies was low (I^2^ = 0%, *p* = 0.58), indicating consistent findings (Figure 5).

#### 3.3.3. Left Ventricular End-Systolic Volume

**Figure 6:**
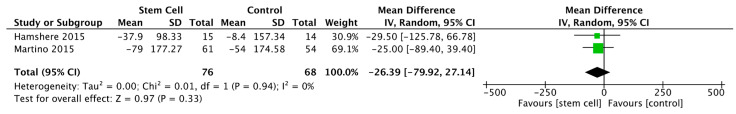
Forest plot for LVESV changes at 12 months.

The left ventricular end-systolic volume was reported in 2 trials, including 144 patients. The combined mean differences in the studies demonstrated no significant difference in LVESV at the 12-month follow-up (MD = −26.39, 95% CI −79.92 to 27.14, *p* = 0.33). The heterogeneity among the studies was low (I^2^ = 0%, *p* = 0.94), indicating consistent findings (Figure 6).

#### 3.3.4. Left Ventricular End-Diastolic Diameter

**Figure 7:**
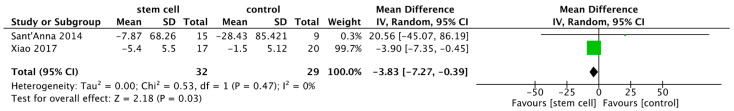
Forest plot for LVEDD changes at 3 months.

The left ventricular end-systolic diameter was reported in 2 trials, including 61 patients. The combined mean differences in the studies demonstrated that the stem cell group improved LVEDD at the 3-month follow-up (MD = −3.83, 95% CI −7.27 to −0.39, *p* = 0.03). The heterogeneity among the studies was low (I^2^ = 0%, *p* = 0.47), indicating consistent findings (Figure 7).

#### 3.3.5. 6-Minute Walking Test

**Figure 8:**
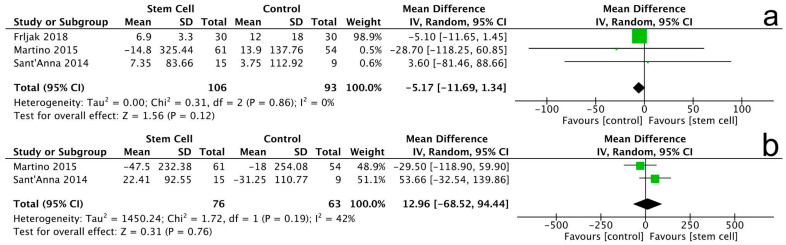
Forest plot for 6-MWT changes at 6 months (**a**) and 12 months (**b**).

A total of 3 trials involving 199 patients evaluated the 6-MWT outcome. At the 6-month follow-up, no statistically significant difference was observed in the 6-MWT results between the stem cell group and the control group (MD = −5.17, 95% CI −11.69 to 1.34, *p* = 0.12). The heterogeneity among the studies was low (I^2^ = 0%, *p* = 0.86), indicating consistent results across the trials. At the 12-month follow-up, the analysis also revealed no significant improvement in the 6-MWT for the stem cell group compared to the control group (MD = 12.96, 95% CI −68.52 to 94.44, *p* = 0.76). The heterogeneity for this follow-up was moderate (I^2^ = 42%, *p* = 0.19) (Figure 8).

#### 3.3.6. New York Heart Association (NYHA) Class

**Figure 9:**
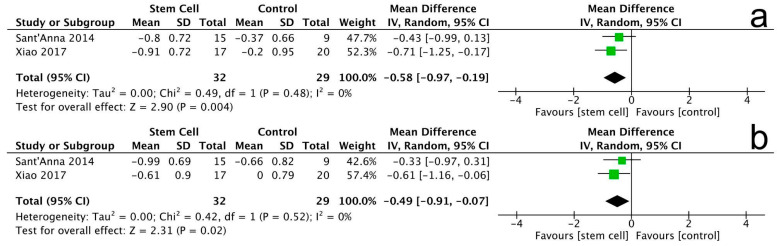
Forest plot for NYHA changes at 3 months (**a**) and 12 months (**b**).

The analysis included two trials assessing the New York Heart Association (NYHA) class with a total of 61 patients. At the 3-month follow-up, there was a significant reduction in the NYHA class for the stem cell group compared to the control group (MD = −0.58, 95% CI −0.97 to −0.19, *p* = 0.004). The heterogeneity among the studies was low (I^2^ = 0%, *p* = 0.48), indicating consistent findings across the trials (Figure 9a).

At the 12-month follow-up, the results remained significant, showing a further reduction in the NYHA class for the stem cell group (MD = −0.49, 95% CI −0.91 to −0.07, *p* = 0.02). The heterogeneity for this follow-up was also low (I^2^ = 0%, *p* = 0.52) (Figure 9b).

#### 3.3.7. Minnesota Living with Heart Failure Questionnaire (MLHFQ)

**Figure 10:**
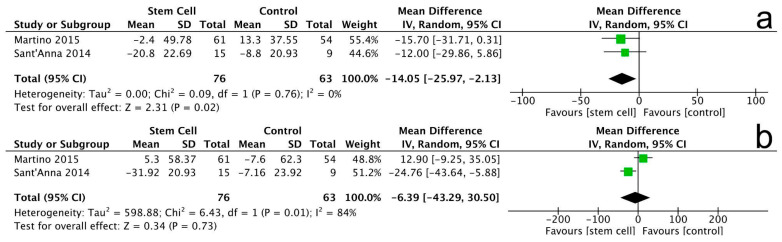
MLHFQ changes at 6 months (**a**) and 12 months (**b**).

Two trials assessed the Minnesota Living with Heart Failure Questionnaire (MLHFQ) outcomes, involving a total of 139 patients. At the 6-month follow-up, there was a significant improvement in MLHFQ scores for the stem cell group compared to the control group (MD = −14.05, 95% CI −25.97 to −2.13, *p* = 0.02), indicating a reduction in the burden of heart failure symptoms. The heterogeneity among the studies was low (I^2^ = 0%, *p* = 0.76) (Figure 10a).

At the 12-month follow-up, the results continued to show a significant improvement in MLHFQ scores for the stem cell group (MD = −6.39, 95% CI −43.29 to 30.50, *p* = 0.73), although the heterogeneity was moderate (I^2^ = 84%, *p* = 0.01) (Figure 10b).

#### 3.3.8. Major Adverse Cardiovascular Events

**Figure 11:**
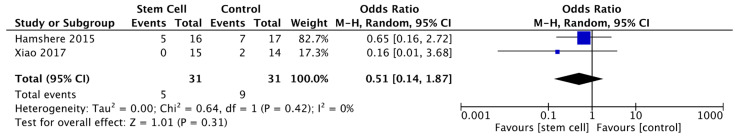
Forest plot for MACEs.

Two trials investigated Major Adverse Cardiovascular Events (MACEs), involving a total of 62 patients. The findings revealed a promising trend indicating that patients receiving stem cell therapy experienced fewer MACEs compared to those in the control group. Specifically, the stem cell group reported five events, while the control group had nine, although the result was not statistically significant (MD = 0.51, 95% CI 0.14 to 1.87, *p* = 0.31). The analysis showed low heterogeneity among the studies (I^2^ = 0%, *p* = 0.42) (Figure 11). Although the results did not achieve statistical significance, they suggest that stem cell therapy may be associated with a reduction in adverse cardiovascular events, highlighting its potential as a beneficial intervention in non-ischemic cardiomyopathy management.

Note: In Figure 4, Figure 5, Figure 6, Figure 7, Figure 8, Figure 9, Figure 10 and Figure 11, the symbols indicate effect sizes for individual studies: squares represent study-specific point estimates and diamond shapes represent pooled effects. Horizontal lines denote 95% confidence intervals. A vertical line represents the line of no effect.

## 4. Discussion

This meta-analysis examining 5 RCTs of 265 patients reveals that stem cell therapy significantly enhances LVEF and LVEDD at 3 months, and an improvement in NYHA functional class was observed at both 3 and 12 months. Additionally, it showed enhanced quality of life (QoL) measured by MLHFQ scores at 6 months. However, we found no significant differences in LVEDV, LVESV, 6-MWT, and MACEs. Our results are consistent with a prior meta-analysis by Tao et al. [25], which reported a significant increase in LVEF and an improvement in functional capacity as evaluated by NYHA class and 6-MWT. Moreover, Kavousi et al. [26] reported significant enhancements in LVEF, NYHA class, MLHFQ scores, and 6-MWT performance after stem cell therapy using MSCs in NICM. Similarly, meta-analyses by Tripathi et al. [27] reported improved LVEF and LVEDD reductions.

In the present analysis, stem cell therapy increased LVEF by 4.55% at the 3-month follow-up and 4.87% at the 12-month follow-up, which is in line with the prior meta-analyses by Tao et al. [25], which reported an improvement in LVEF of 4.84%, and Tripathi et al. [27], which reported an improvement in LVEF of 4.17% on average. The majority of studies utilized the administration route of injection via the intracoronary route using CD34^+^-type cells derived from Bone Marrow Mononuclear Cells (BMMCs) and Bone Marrow Mesenchymal/Stromal Cells (BMSCs). Similar to those studies, most trials in our analysis employed intracoronary delivery of CD34^+^ cells derived from BMMCs or BMSCs. CD34^+^ cells, which are enriched in endothelial progenitor cells, exert their effect primarily through paracrine signaling, which secretes growth factors, promotes microvascular angiogenesis and antifibrotic remodeling, and enhances myocardial contractility [25,26,27]. According to clinical evidence from Vrtovec et al. [28,29], transendocardial injection of CD34^+^ cells in NICM patients has shown improvements in LVEF and enhanced exercise capacity. Although CD133^+^ cells have been less frequently investigated, Sant’Anna et al. [20] reported improved LVEF, NYHA class, and exercise capacity following intracoronary CD133^+^ cell administration. Recent meta-analyses also indicate that intracoronary delivery may yield superior outcomes compared with transendocardial or intramyocardial routes [20,30]. Unlike previous studies, which often include mixed cardiomyopathy populations, the present meta-analysis focused exclusively on non-ischemic cardiomyopathy (NICM) and incorporated only randomized controlled trials. By comparing outcomes across different cell types, delivery routes, and follow-up durations, this study provides new insight into the specific efficacy and durability of stem cell therapy in NICM.

**Figure 12:**
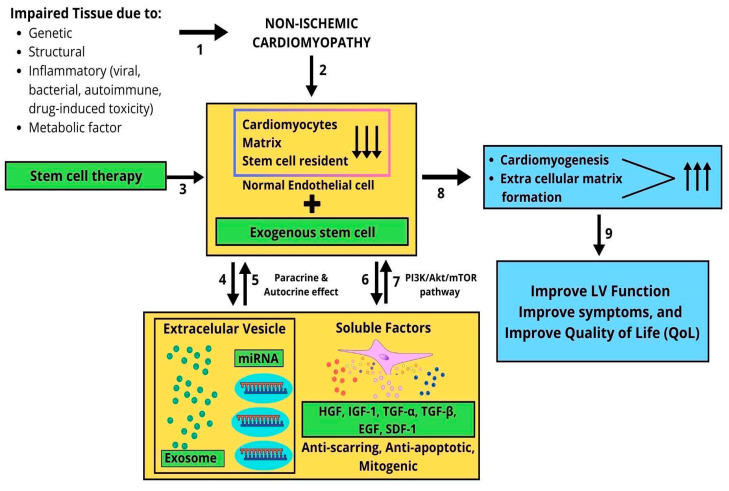
How stem cell therapy in NICM improves outcomes. Proposed mechanism of stem cell therapy in non-ischemic cardiomyopathy (NICM): (1) genetic, structural, inflammatory, or metabolic factors cause myocardial injury; (2) damage leads to cardiomyocyte loss, extracellular matrix disruption, and depletion of resident stem cells; (3) exogenous stem cells are delivered via intracoronary, transendocardial, or intramyocardial routes; (4,5) transplanted cells exert paracrine and autocrine effects through extracellular vesicles and microRNA; (6,7) secretion of soluble factors (HGF, IGF-1, TGF-α/β, EGF, and SDF-1) activates PI3K/Akt/mTOR signaling, promoting anti-apoptotic and anti-fibrotic actions; (8) the effects enhance cardiomyogenesis and extracellular matrix formation; (9) overall, left ventricular function, symptoms, and quality of life (QoL) improve in NICM patients.

Theoretically, these improvements can be achieved because stem cells primarily exert benefits via paracrine signaling [31]. The heart consists of cardiomyocytes, endothelial cells, and fibroblasts, which communicate primarily through paracrine signaling. Paracrine signaling is essential for sustaining cardiac physiology and ensuring myocardial adaptation under stressors (Figure 12). This mechanism plays diverse roles in reverse remodeling of the heart, meaning it is able to facilitate angiogenesis, cardiomyogenesis, and matrix formation to maintain heart integrity [31,32]. Cardiac cells release factors that activate not only paracrine but also autocrine signaling. Autocrine pathways play diverse physiological roles that support the preservation of cardiac structure and function under both ischemic and non-ischemic conditions [31]. Paracrine and autocrine abilities have led to a great deal of interest in their use as stem cell therapeutic agents for tissue repair and regeneration.

The reduction in myocardial function due to genetic, structural, inflammatory, and metabolic factors leads to a reduction in cardiomyocytes, extracellular matrix components, and resident stem cells. In the context of non-ischemic cardiomyopathy (NICM), cardiomyocytes undergo paracrine and autocrine signaling processes, secreting exosomes containing cardiac-specific microRNAs (miRNAs). These exosomes aim to produce soluble factors—such as hepatocyte growth factor (HGF), insulin-like growth factor-1 (IGF-1), transforming growth factor α/β (TGF-α/β), epidermal growth factor (EGF), and stromal cell-derived factor 1 (SDF-1)—which function as anti-apoptotic, anti-fibrotic, and mitogenic agents. The PI3K/AKT/mTOR signaling pathways mediate the action of these soluble factors, promoting cardiomyocyte proliferation, extracellular matrix remodeling, and the proliferation and differentiation of both resident and exogenous stem cells to facilitate cardiomyogenesis and the formation of new matrix components. Stem cell therapy has been shown to improve left ventricular (LV) function, alleviate symptoms, and enhance quality of life (QoL) (Figure 12).

Stem cell therapy studies in NICM are less common compared to ICM because the myocardial regeneration concept is more easily applied, as it usually presents with a well-defined area of myocardial loss and scar tissue, in contrast with NICM, which often involves diffuse myocardial fibrosis [25]. Evidence from head-to-head comparisons suggests that the response to stem cell therapy differs between NICM and ICM, but this is still poorly understood [25,27]. Several studies found that MSCs work based on myocardium viability. NICM has more viable myocardium compared to ICM, leading MSCs to primarily act by reducing fibrosis and improving myocardial contractility, in contrast to ICM, where MSCs prioritize structural regeneration by neovascularization scarred ischemic tissue. Paracrine signaling also plays a crucial role in NICM by enhancing the surviving myocyte performance of viable myocytes [32]. In NICM, the higher proportion of viable myocardium and less dense scar tissue appears to favor improvements in systolic function, reflected in greater gains in LVEF and stroke volume [16,17]. Meanwhile, ICM is characterized by large regions of non-viable scarring, which have more pronounced benefits in angiogenesis and scar stabilization due to paracrine-driven angiogenesis and extracellular matrix modulation in border zones [15,16,17].

From a clinical perspective, our findings indicate that stem cell therapy is considered an adjunctive strategy to improve symptoms and quality of life in NICM [17,28]. Even though NICM is not as common as ICM in stem cell therapy, these findings provide evidence that stem cell therapy shows potential improvement in cardiac function, though evidence of safety and reverse remodeling remains limited due to small sample sizes. NICM may be valuable in the future for NICM therapy as an adjunct to GDMT or device therapy.

Although the pooled analysis demonstrated improvements in LVEF, NYHA functional class, and quality of life, these results should be interpreted with caution. As the small number of studies and patients influenced a number of important outcomes, including major adverse cardiovascular events (MACEs), left ventricular sizes, and LVEDD, the findings had low statistical power and were not as reliable. Therefore, rather than an actual lack of effect, the apparent lack of a meaningful difference in ventricular remodeling parameters may be the result of an insufficient sample size. There was a notable improvement in LVEF at 3 months, but it seemed less noticeable at 6 and 12 months. This could be because there were not many studies that reported longer-term follow-up, rather than a real loss in therapeutic efficacy.

This review has several limitations that should be acknowledged. First, the number of available RCTs in NICM remains limited, with relatively small sample sizes, which may reduce the statistical power of the results. Second, there was heterogeneity in the stem cell types and delivery routes, which could influence treatment outcomes. Third, the follow-up duration in most included studies was relatively short, limiting our ability to assess the long-term safety. Further large-scale trials are needed to assess the long-term efficacy of stem cell therapy in NICM. Finally, publication bias cannot be entirely excluded given the small pool of studies and the possibility that negative results are underreported.

## 5. Conclusions

Bone-marrow-derived stem cell therapy could be a promising and safe method to improve cardiac function and quality of life in patients with NICM. Further large-scale randomized controlled trials are needed to validate and strengthen these findings.

## Figures and Tables

**Table 1:** Study characteristics.

No	First Author Name, Year	Study Design	Country of Origin	Number of Participants	Type of Stem Cell	Dose of Cells Injected (n)	Route of Administration	Duration of Follow-Up	Outcome of Interest
1	Sant’Anna RT et al., 2014 [20]	RCT	Brazil	30	BMMC	1.06 ± 0.43 × 10^8^	Intramyocardial, transthoracic transplantation	3 months & 9 months	LVEF, LVESV, LVEDV, 6-MWT, NYHA, MLHFQ
2	Hamshere S et al., 2015 [21]	RCT	UK	60	BMSC	216.0 ± 221.8 × 10^6^	Intracoronary infusion	3 months & 12 months	LVEF, LVESV, LVEDV, NYHA, NT-proBNP, MACE
3	Martino H et al., 2015 [22]	RCT	Brazil	115	BMMC	8 × 10^7^	Intracoronary injection	6 months & 12 months	LVEF, LVESV, LVEDV, NYHA, 6-MWT, MLHFQ
4	Xiao W et al., 2017 [23]	RCT	China	37	BMMC	5.1 ± 2.0 × 10^8^	Intracoronary infusion	3 months and 12 months	LVEF, MPD, LVEDd, NYHA, MACE
5	Frljak S et al., 2018 [24]	RCT	USA	60	CD34^+^	8 × 10^7^	Transendocardial	6 months	LVEF, TAPSE, NT-proBNP, 6-MWT, LVEDd

BMMC: bone marrow mononuclear stem cell; BMSC: bone marrow-derived mesenchymal/stromal cell; LVEF: left ventricular ejection fraction; LVESV: left ventricular end-systolic volume; LVEDV: left ventricular end-diastolic volume; 6-MWT: 6-min walking test; NYHA: New York heart association; MLHFQ: Minnesota living with heart failure questionnaire; NT-proBNP: N-terminal pro–B-type natriuretic peptide; MACE: major adverse cardiovascular events; MPD: myocardial perfusion defects; LVEDd: left ventricular end-diastolic diameter; TAPSE: tricuspid annular plane systolic excursion.

## Data Availability

The original contributions presented in this study are included in the article/Supplementary Materials. Further inquiries can be directed to the corresponding author.

## Supplementary Materials

The following supporting information can be downloaded at: https://www.mdpi.com/article/10.3390/jcm14217610/s1, PRISMA 2020 Checklist.

## Abbreviations

The following abbreviations are used in this manuscript:

NICM	Non-ischemic cardiomyopathy
ICM	Ischemic cardiomyopathy
CT-scan	Computed tomography scan
MRI	Magnetic resonance imaging
DCM	Dilated cardiomyopathy
HCM	Hypertrophic cardiomyopathy
RCM	Restrictive cardiomyopathy
ARVC	Arrhythmogenic right ventricular cardiomyopathy
NDLVC	Non-dilated left ventricular cardiomyopathy
GBD	Global burden of disease
AHA	American Heart Association
ESC	European Association of Cardiology
DALYs	Disability-adjusted life years
WHF	The world heart federation
PRISMA	Systematic Reviews and Meta-Analyses
RCTs	Randomized controlled trials
LVEF	left ventricular ejection fraction
LVEDV	left ventricular end-diastolic volume
LVESV	left ventricular end-systolic volume
NYHA	New York Heart Association
6MWT	6-min walk distance test
MACE	major cardiac adverse events
